# Distraction Enterogenesis in Rats: A Novel Approach for the Treatment of Short Bowel Syndrome

**DOI:** 10.3390/pathophysiology31030029

**Published:** 2024-07-30

**Authors:** Collyn O’Quin, Sean D. Clayton, Lexus Trosclair, Hannah Meyer, Nhi H. Dao, Andrew Minagar, Luke White, Valerie Welch, Giovanni Solitro, Jonathan Steven Alexander, Donald Sorrells

**Affiliations:** 1 Department of Surgery, LSU Health Shreveport, Shreveport, LA 71103, USA; cco001@lsuhs.edu (C.O.); sean.clayton@lsuhs.edu (S.D.C.); lexus.trosclair@lsuhs.edu (L.T.);; 2 Department of Molecular and Cellular Physiology, LSU Health Shreveport, Shreveport, LA 71103, USAamina2@lsuhsc.edu (A.M.); luke.white@lsuhs.edu (L.W.); jonathan.alexander@lsuhs.edu (J.S.A.); 3 Department of Pathology, LSU Health Shreveport, Shreveport, LA 71103, USA; valerie.welch@lsuhs.edu; 4 Department of Orthopedic Surgery, LSU Health Shreveport, Shreveport, LA 71103, USA; giovanni.solitro@lsuhs.edu

**Keywords:** intestinal expansion sleeve, distraction enterogenesis, short bowel syndrome, necrotizing enterocolitis, intestinal elongation

## Abstract

Background: Surgeons often encounter patients with intestinal failure due to inadequate intestinal length (“short bowel syndrome”/SBS). Treatment in these patients remains challenging and the process of physiologic adaptation may take years to complete, which frequently requires parenteral nutrition. We propose a proof-of-concept mechanical bowel elongation approach using a self-expanding prototype of an intestinal expansion sleeve (IES) for use in SBS to accelerate the adaptation process. Methods: IESs were deployed in the small intestines of Sprague Dawley rats. Mechanical characterization of these prototypes was performed. IES length–tension relationships and post-implant bowel expansion were measured ex vivo. Bowel histology before and after implantation was evaluated. Results: IES mechanical studies demonstrated decreasing expansive force with elongation. The deployment of IES devices produced an immediate 21 ± 8% increase in bowel length (*p* < 0.001, *n* = 11). Mechanical load testing data showed that the IESs expressed maximum expansive forces at 50% compression of the initial pre-contracted length. The small-intestine failure load in the rats was 1.88 ± 21 N. Intestinal histology post deployment of the IES showed significant expansive changes compared to unstretched bowel tissue. Conclusions: IES devices were scalable to the rat intestinal model in our study. The failure load of the rat small intestine was many times higher than the force exerted by the contraction of the IES. Histology demonstrated preservation of intestinal structure with some mucosal erosion. Future in vivo rat studies on distraction enterogenesis with this IES should help to define this organogenesis phenomenon.

## 1. Introduction

Short bowel syndrome (SBS) is a multisystemic disorder that results in intestinal failure, usually as the result of necrotizing enterocolitis, malrotation with volvulus, intussusception, or idiopathic causes [1]. Pediatric surgeons most often encounter patients with intestinal failure due to inadequate intestinal length [2]. These patients are challenging because bowel adaptation and enteral absorption remain low [3]. The length of small bowel required for adequate absorption is widely discussed; however, a bowel length less than 100 cm in the first year of life is abnormal and lengths less than 40 cm often require additional therapies [4]. Patients with SBS compensate through gastroparesis and slow dilation of the intestinal diameter [5]. This process may take months, or years, requiring supplemental parenteral nutrition for adequate growth [6]. Although parenteral nutrition is necessary, its supplementation can pose several significant hazards. These include central line-associated bloodstream infections (CLASBIs) and parenteral-associated liver disease [7]. Additionally, parenteral nutrition for SBS poses significant costs that are estimated to be greater than USD 2 billion per year in the United States alone [8].

Bowel-lengthening procedures have been implemented for treatment of SBS as an alternative to small-intestine transplantation. Autologous lengthening procedures offer the benefit of using one’s own bowel and avoid complicated, expensive techniques [9]. Surgical intervention such as the Bianchi procedure [10] and serial transverse enteroplasty (“STEP”) [11] have been implemented to increase the length of the native bowel. This intestinal lengthening results in earlier weaning from parenteral nutrition in select patients [12,13], but these surgical techniques are limited by the need for a sufficiently dilated bowel diameter [14]. To overcome this limitation, distraction enterogenesis (DE) has been more recently proposed as a means to provide sufficient bowel elongation to decrease parenteral nutrition needs. Investigations into the application of distraction forces to bones have shown effective “osteodistraction” in long bone reconstruction [15]. Building on this technique, distraction organogenesis has been applied in several more recent investigations for treating SBS. In early investigations on intestinal distraction, extraluminal expanders placed on the antimesenteric side of rabbit small bowels were found to result in effective bowel lengthening [16]. Subsequent investigations explored intraluminal techniques for intestinal distraction. For example, a hydraulic piston system was designed and used to significantly change the length of the rabbit small intestine, resulting in an increase in mucosal thickness, crypt depth, and epithelial cell proliferation [17]. Further investigation was performed on rat small intestines using an externally placed intraluminal expander, which also showed a significant increase in bowel length and successful enterogenesis [18]. While mechanical characterization of intestinal expansion sleeves (IESs) has been performed in rabbits [19], a scaling of this technique for rats has not been performed. Ex vivo IES testing in the rat model would therefore assist in design evaluation. Ex vivo experimentation in the rat model will allow us to rigorously control experimental variables and directly observe the mechanical effects of the expansive device on intraluminal intestinal tissue. This approach minimizes confounding variables such as systemic physiological responses or peristaltic movement of the intestine, thus providing a clear understanding of the device’s fundamental mechanics. Therefore, we aim to characterize the IES in terms of distraction force and verify its capacity for intestinal distraction in a rat model. We hypothesize that a properly scaled IES can achieve significant intestinal distraction without inducing intestinal failure in an ex vivo rat model.

## 2. Materials and Methods

This study was approved by the IACUC (Institutional Animal Care and Use Committee) for Louisiana State University Health Sciences Center in Shreveport. This study was executed in three phases: (1) we quantified the device distraction force exercised in relation to compression; (2) we evaluated ex vivo the suitable distraction load for the small intestine; and (3) we performed a quantitative measure of distraction coupled with a qualitative histological analysis to verify the microscopic changes caused by internal intestinal distraction.

### 2.1. IES Distraction Force Characterization

**Figure 1:**
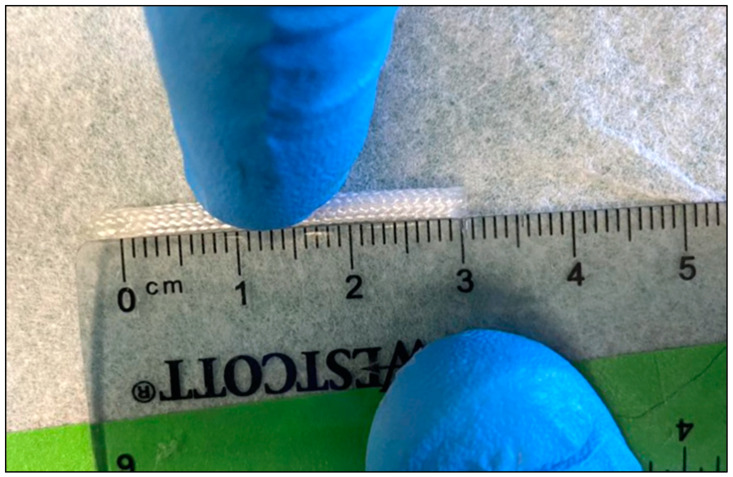
IES device with angled (**left**) and sealed (**right**) ends measured to a 30 mm nominal length.

Intestinal expansion sleeves (IESs) were created from 2 mm proprietary braided composite with helicoid trusses and isometric ends [19]. Three centimeters of this sleeve material was measured with calipers and cut to size. One end of the IES was sealed with a barium–liquid rubber sealant mixture to both prevent the fraying of the ends of the IES device during compression and allow easy visualization of the device postoperatively. The other side was cut at approximately a 45° angle to facilitate easier insertion into the intestinal lumen. The IES device is shown in Figure 1.

**Figure 2:**
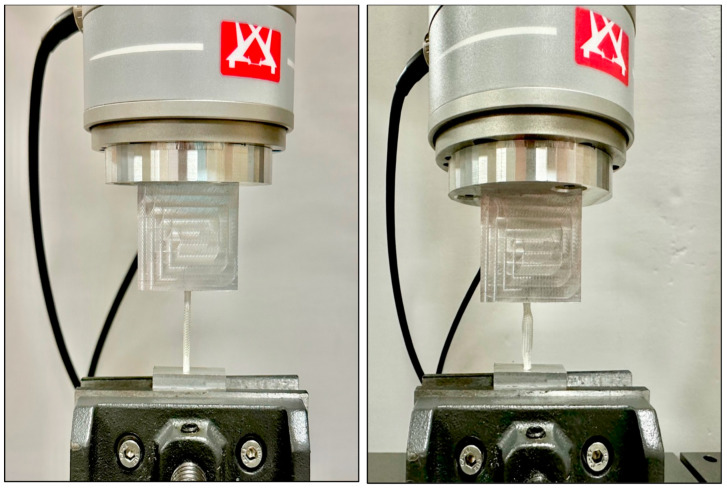
Mechanical characterization of IES device at i.l. (**left**) and 50% compressed (**right**).

Evaluation of the distraction force provided by the IES devices at various compression states was performed using an Instron 8872 Servo hydraulic testing system (Instron, Norwood, MA). Compression was applied followed by distraction at a rate of 50 mm/min to determine the forces that the sleeves expressed at 50%, 40%, 30%, 20%, and 10% compression (Figure 2).

### 2.2. Small-Bowel Characterization 

**Figure 3:**
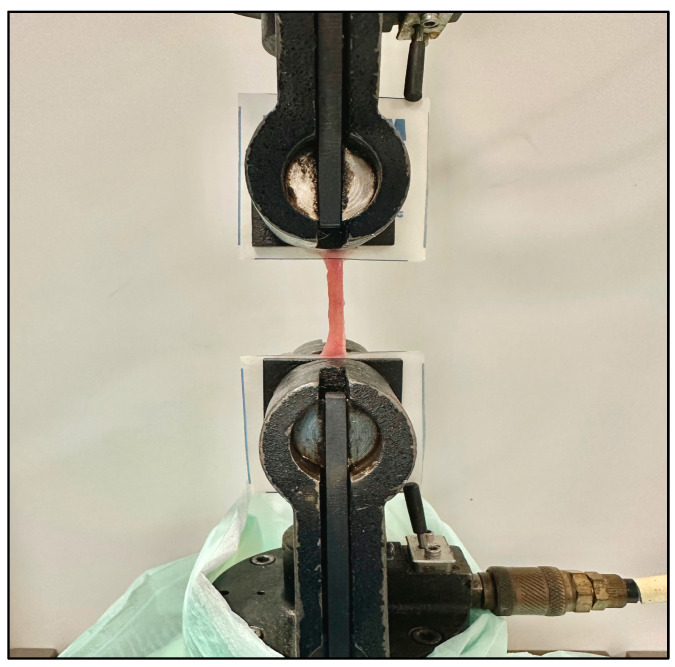
Mechanical characterization of native small intestine, measuring the expansive force required to produce intestinal failure.

Native rat small intestine was harvested for mechanical characterization to determine the maximum expansive force that the intestine can withstand prior to failure. Intestinal segments from control rats were harvested by first identifying the small bowel; then, sections of jejunum were harvested. Once the target tissue was identified, harvesting of the tissues was performed using Metzenbaum scissors. The tissue was immediately contained in a cool, saline-moistened Petri dish for short-term preservation to prepare for mechanical testing. The ends of the intestinal segments were placed between two pieces of parafilm and anchored using a staple. This would allow sufficient support for the pneumatic clamps to adequately grasp the tissue during distraction. Testing was performed using the same Instron 8872 system used for IES characterization (Figure 3). Each end of the native bowel was secured between parafilm and held in place between two pneumatic grips at 25 psi. Displacement was imposed at a rate of 50 mm/min until failure; force was recorded at a frequency of 100 Hz.

### 2.3. Ex Vivo Deployment and Histological Analysis 

**Figure 4:**
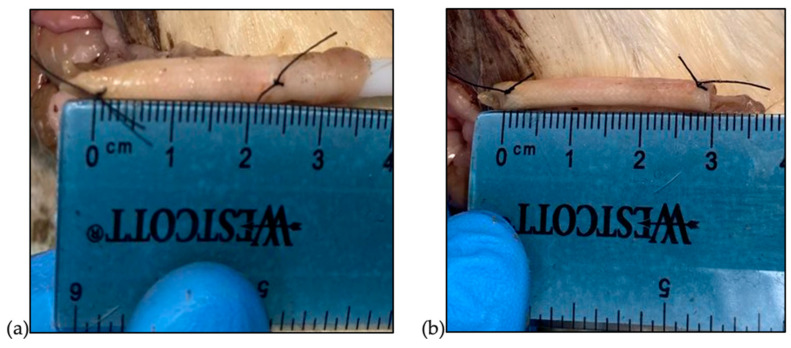
IESs inserted and sutured in the pre-deployment contracted state (**a**) and the post-deployment state (**b**).

The IES devices were implanted in the small intestine of euthanized Sprague Dawley rats. Bowels were harvested and washed with isotonic saline. The IES devices were compressed over plastic dilators prior to insertion. Contracted IES devices were inserted into the segment of the intestinal lumen. While still contracted, 6-0 silk sutures were placed at four equidistant points at each end to secure the device to the small intestine in the pre-contracted state and the contracted length was measured (Figure 4). The device was anchored to the intraluminal intestinal wall via the four sutures passed from the extraluminal side of the intestine, through the sleeve, and back out the extraluminal side where it was secured with a surgeon’s knot. Since this experiment was conducted ex vivo, this method of securing the device was feasible. Once secured, the dilator was removed, and the device was allowed to expand to its post-deployment length (Figure 4). Following the deployment of the device, the tissue was collected from the rats and fixed in 10% phosphate-buffered formalin. Following overnight fixation and processing, tissues were embedded in paraffin and sectioned onto glass slides. Routine hematoxylin and eosin staining was performed. Slides were reviewed and photographs were taken by a board-certified anatomic pathologist.

### 2.4. Statistical Analysis

The distraction load characterizing the device and the distraction achieved in the rat models were both analyzed for normality using the Shapiro–Wilk test and differences were analyzed using Student’s *t*-test or the equivalent nonparametric Wilcoxon signed rank test at a significance level of 0.05. Additionally, a power analysis for the ex vivo IES deployment was determined using a two-sample *t*-test.

## 3. Results

### 3.1. IES Distraction Force

Mechanical load testing was performed on the IES devices with an average nominal length of 30.3 mm, and expansive force was recorded at 50%, 40%, 30%, 20%, and 10% compression of the IES device (Table 1). Interestingly, expansive forces were highest at 50% compression, with a steep drop in force as the device extended towards its initial pre-contracted length.

### 3.2. Bowel Failure Load 

In addition to mechanical characterization of the IES devices, the excised rat small intestine was mechanically characterized to determine the force causing failure (tearing) of the intestines. Three intestinal segments were tested and showed failure at 1.88 ± 21 N.

### 3.3. Ex Vivo Deployment and Histology

Eleven IES devices were implanted post mortem into Sprague Dawley rat small intestines. Pre-contracted IES devices were placed into the portion of the small intestine at 72 ± 5% of their initial length (i.l.). Deployment of the IES devices produced significant lengthening of the small intestine to 92 ± 7% i.l., resulting in an immediate distraction of 21 ± 8% of the small intestine (*p* = 0.00365, n = 11).

**Figure 5:**
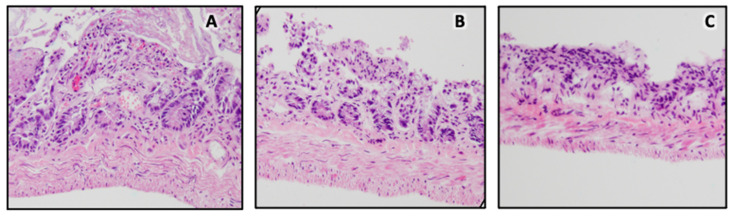
Histological analysis of rat intestinal tissue. (**A**) Control tissue; (**B**,**C**) stretched tissue after IES deployment, demonstrating significant thinning of the muscularis and residual mucosa.

A qualitative analysis of the expanded small-intestine tissue was performed. Histologic evaluation of the control tissues revealed significant autolysis affecting primarily the mucosal layer but up to the entire small-intestine wall in some foci. No significant tissue damage was noted. Histologic evaluation of the tissues post ex vivo deployment revealed some potential autolysis, which affected the mucosal layer in some regions. No pre-existing enteritis, increased inflammation, or other significant pathology was seen. While the tissue integrity post ex vivo deployment was maintained overall, microscopic stretching of muscle fibers was noted in all five post-ex-vivo-deployment tissue samples. There were also many foci of complete or near-complete mucosal loss produced by the introduction/deployment of the device (Figure 5).

## 4. Discussion

Inadequate intestinal function for complete enteral nutrition is a significant cause of morbidity and mortality. Infants and children are disproportionately affected due to their need for growth velocity [16]. The process of adaptation of the residual intestine is a slow process involving dilation of the bowel diameter to increase the villus surface area for absorption. Patients also develop gastroparesis to limit transit through the stomach. The intestinal adaptation process rarely results in increases in length. Independence from parenteral nutrition is directly correlated with intestinal length [17]. In the current study, we were able to successfully achieve immediate intestinal distraction while maintaining continuity of the intestine. Furthermore, the IES devices under compression loads less than 50% exhibited a longitudinal force that was smaller than the determined intestinal failure load, virtually eliminating the risk of bowel rupture from device implantation.

Our study tested the force required to disrupt the rat intestine (1.88 ± 0.21 N). Devices used for distraction enterogenesis will need to exert a fraction of this disruptive force on the intestine. The “goldilocks” zone of force that causes useful distraction enterogenesis has not yet been defined. Too little force will not cause enough elongation to be significant and too much would be disastrous with intestinal perforation. Our sleeves exerted a force of 0.22 ± 0.01 N, which caused significant elongation ex vivo. In vivo studies will be needed to validate the benefits of IESs.

**Figure 6:**
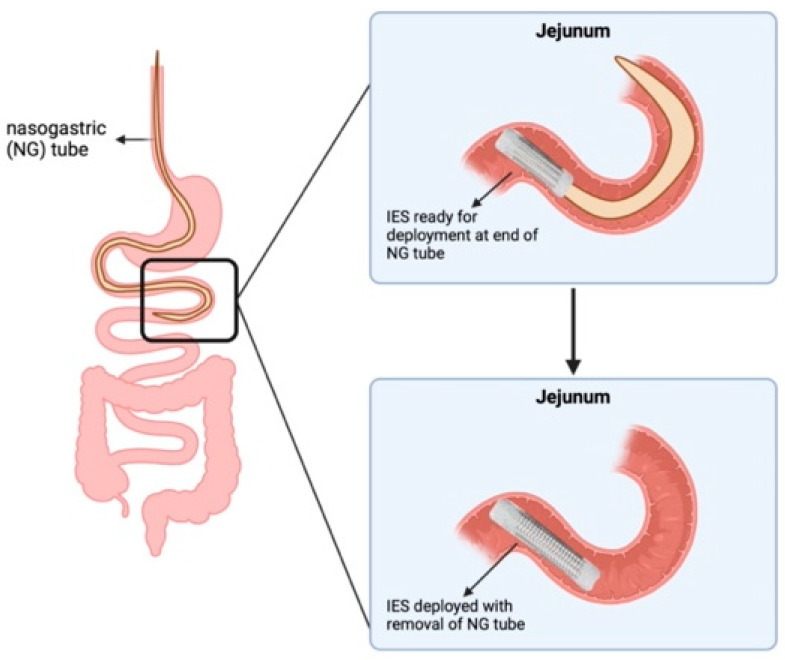
Potential deployment strategy for future in vivo IES implantation.

In the current study, the rat IES device was able to exert a force of 0.22 N, significantly lower than the rat intestinal failure load. The IES device produced an initial 21% elongation of the jejunal segment. Despite being noteworthy, the maximum potential distraction achievable was restricted by the length of the IES device. Our ability to attain length was constrained by the sleeve’s capacity to expand up to its initial length. For comparison, in another intestinal distraction study on a murine model, an external intestinal elongation device that was increased at a rate of 1 mm per day resulted in an 149% increase in intestinal length [18]. Additionally, a variety of devices have been used for distraction enterogenesis [19,20,21,22]. Most of these are placed in the intestine, but there have been attempts at external devices [21]. External devices would require several surgeries and may be prone to mechanical complications like obstruction, device dislodgement and migration, and erosion. Dunn et al. used a nitinol spring to cause bowel elongation [22]. This spring must be “fixed” in place with sutures or vessel loops. These devices did significantly lengthen intestines [22,23,24,25]. In comparison, our device is a woven mesh sleeve that increases in diameter slightly with the shortening of the sleeve (contraction). Clinically, the increase in diameter could be used to secure the sleeve in a segment of bowel. The sleeve would need a “clinging” mechanism that would secure it to the bowel when compressed. This “clinging” mechanism could be achieved using bio-inspired hooks added to the design of the IES or expansive stents like those used for endovascular repair of vessels. The deployment of our sleeve could be achieved in several ways, such as with a transoral long nasogastric tube with a sleeve attached to the end or with the IES on the end of an endoscope that can be passed through a stoma (Figure 6). The sleeve also offers significant surface area for drug attachment. However, a limitation of this study is that the characterization was based on a convenience sample of only three segments. Additionally, the sample size was not determined through a power analysis, potentially affecting the generalizability of the findings.

Our histologic sections demonstrated expected thinning of the layers of the intestine, which is anticipated because of the stretching of the tissues induced by the introduction of the device. Autolysis was seen upon histologic evaluation and was found in both the control and test tissues, which reflects the brief time that elapsed between rat sacrifice, device placement, and tissue collection/placement in formalin. The device was deployed in the small-intestine tissue ex vivo, and inflammation was not expected. Because the tissue was placed into formalin immediately following device deployment, autolysis may have already occurred prior or concurrent with device deployment. Autolysis could have made the mucosa more vulnerable to physical/extrinsic insults, which may explain the degree of mucosal denudation that followed device deployment. The stretching of muscle fibers post device deployment is consistent with the tissue stretching caused by the device. The thinning of intestinal layers would almost certainly be a transient phenomenon, as noted by other investigators in vivo. Dunn et al. demonstrated a thickening of the muscularis mucosa using their spring distraction device [25]. Other in vivo studies have also shown a significant decrease in the numbers of ganglion cells in submucosa via immunohistochemical staining [26]. The effect of these changes on function appears to be minimal [27,28]. Future in vivo studies are necessary to evaluate the tissue response and integrity post device deployment, since well-oxygenated, intact small-intestine tissue is expected to withstand both physical and chemical insults.

One goal of intestinal distraction using the IES is to achieve the significant bowel length necessary to decrease supplemental parenteral nutrition. The specific distraction length required to achieve autonomy from parenteral nutrition and thus clinical improvement of SBS remains variable depending on the patient. One study found that adults with SBS require a bowel length (cm)-to-body weight (kg) ratio of 1.0 cm/kg in order to become independent of parenteral nutrition; patients with a ratio between 0.5 and 1.0 have a higher probability (80%) of achieving independence from parenteral nutrition and patients with a ratio of <0.5 have about a 50% chance of achieving independence [29]. This ratio provides a quantitative value to help determine the predicted amount of intestinal distraction that must be met using the IES device. Although the current study demonstrates significant intestinal distraction, future studies will probably require multiple deployments of IES devices to achieve the desired bowel length-to-body weight ratio needed for nutritional autonomy and decreased use of parenteral nutrition.

Our study shows that immediate intestinal distraction is achievable with our IES device. This study was performed ex vivo on rat small intestines. Although immediate longitudinal intestinal distraction was obtained, we were unable to characterize the changes in intestinal lengthening over time. However, another study showed that epithelial proliferation and muscularis thickening occurs after expansion using a hydraulic lengthening system [30]. This demonstrates that distraction enterogenesis is attainable and that our IES devices would likely provide similar results. Moreover, the utilization of ex vivo intestine samples from expired rat models presented an additional limitation in this investigation. The degree of autolysis observed during histological evaluation of the distracted tissues raised uncertainty about whether it directly resulted from IES implantation. To address this concern comprehensively, future studies using live Sprague Dawley rats will be forthcoming, enabling us to evaluate in vivo distraction enterogenesis and thoroughly assess histological changes secondary to IES. Forthcoming investigations will delve into IES deployment and reproducibility, aiming to precisely quantify the extent of distraction achieved through enterogenesis. Subsequent phases of IES development and application are envisioned to incorporate innovative techniques, such as integrating a biodegradable stylet for maintaining the IES in a compressed configuration. Furthermore, more avenues for exploration encompass embedding the IES within absorbable biomaterials like polyvinyl alcohol (PVA), or, alternatively, implementing cross-linked PVA-embedded IES structures. This strategic approach would facilitate controlled contraction and gradual expansion of the IES, mitigating the potential risk of tissue damage while achieving sustained therapeutic benefits.

While this study provides valuable insights, several limitations should be considered. First, the ex vivo nature of the studied model, while allowing for controlled experimentation, may not fully capture the complex physiological interactions present in vivo, thus affecting the direct clinical applicability of our results. Additionally, the sample processing in formalin, necessary for elongation measurement and histological analysis, can introduce artifacts and shrinkage, potentially impacting the precision of these assessments. The small sample size, with only three rats being measured, also limits the statistical power and generalizability of the bowel failure load results. Despite these constraints, our findings offer a foundational understanding that can inform future research. Moreover, the ex vivo model does not account for dynamic factors such as peristaltic movements, anastomotic integrity, and inflammation, which are crucial for a comprehensive understanding of bowel function in living organisms. These limitations highlight areas for future studies, particularly those involving larger sample sizes and in vivo models, to build on the promising results presented here and enhance their clinical relevance.

## 5. Conclusions

The IES represents a promising platform to obtain longitudinal bowel elongation via distraction enterogenesis in SBS. Our IES results demonstrate intestinal lengthening with a distractive force tailored to the rat small intestine. Optimal intestinal lengthening may require multiple IES devices to achieve independence from parenteral nutrition. These devices may ultimately be placed without surgery on the end of a long feeding tube.

## Figures and Tables

**Table 1:** IES’s exercised distraction force at various compression states.

Device Compression	Average Expansive Force [N]
50%	2.80 ± 0.36
40%	0.36 ± 0.02
30%	0.22 ± 0.01
20%	0.13 ± 0.01
10%	0.06 ± 0.01

## Data Availability

The data presented in this study are available on request from the corresponding author.
